# Testing the mate-choice hypothesis of the female orgasm: disentangling traits and behaviours

**DOI:** 10.3402/snp.v6.31562

**Published:** 2016-10-25

**Authors:** James M. Sherlock, Morgan J. Sidari, Emily Ann Harris, Fiona Kate Barlow, Brendan P. Zietsch

**Affiliations:** 1 School of Psychology, University of Queensland, Brisbane, Queensland, Australia; 2 Menzies Health Institute Queensland and School of Applied Psychology, Griffith University, Brisbane, Australia; 3 Genetic Epidemiology Laboratory, Queensland Institute of Medical Research, Queensland, Australia

**Keywords:** evolution, sex, partner choice, mating, relationships

## Abstract

**Background:**

The evolution of the female orgasm in humans and its role in romantic relationships is poorly understood. Whereas the male orgasm is inherently linked to reproduction, the female orgasm is not linked to obvious reproductive or survival benefits. It also occurs less consistently during penetrative sex than does the male orgasm. Mate-choice hypotheses posit that the wide variation in female orgasm frequency reflects a discriminatory mechanism designed to select high-quality mates.

**Objective:**

We aimed to determine (1) whether women report that their orgasm frequency varies between partners, (2) whether this variation reflects mates' personal characteristics, and (3) whether this variation reflects own and partner sexual behaviour during intercourse.

**Design:**

We collected survey data from 103 women who rated (1) the extent to which their orgasm frequency varied between partners, (2) the characteristics of previous sexual partners who induced high-orgasm frequency and those who induced low-orgasm frequency, and (3) the specific behaviours during sex with those partners. This is the first study to test within-woman variation in orgasm and partner traits.

**Results:**

Overall, women reported variation in their orgasm rates with different partners. Partners who induced high-orgasm rates were rated as more humorous, creative, warm, faithful, and better smelling than partners who induced low-orgasm rates, and also engaged in greater efforts to induce partner orgasm.

**Conclusions:**

Some assumptions and predictions of mate-choice hypotheses of female orgasm were supported, while other aspects of our findings provide reasons to remain sceptical.

The evolutionary basis of the female orgasm in humans is poorly understood (Amundson, 2008; Barash, 2005; Barash & Lipton, 2009; Judson, 2005; Puts & Dawood, 2006; Puts, Dawood, & Welling, 2012; Zietsch & Santtila, 2012). Whereas the male orgasm is linked to ejaculation, the function (if any) of the female orgasm is unknown. Different women vary greatly in the ease with which they reach orgasm during sex, partly due to genetic differences (Zietsch, Miller, Bailey, & Martin, 2011), and there are no clear fitness consequences of this variation (Zietsch & Santtila, 2013). However, some researchers have proposed that the female orgasm serves a discriminatory function in mate selection (Alcock, 1980; Puts, Dawood, et al., 2012; Smith, 1984; Thornhill, Gangestad, & Comer, 1995). In this paper, we test some of the core assumptions and predictions of this type of hypothesis.

In essence, mate-choice hypotheses are based on the proposition that due to the high gestational cost of pregnancy, as well as the ongoing cost of rearing children, it is important for women to reproduce with a mate of high quality – that is, one who will offer benefits to the woman and/or her offspring. The nature of those benefits distinguishes two versions of mate-choice hypotheses: the sire-choice hypothesis, which proposes that female orgasm functions to help select mates who will provide genetic benefits to the offspring (Alcock, 1980; Baker & Bellis, 1993; Puts, Welling, Burriss, & Dawood, 2012; Smith, 1984; Thornhill et al., 1995); and the pair-bond hypothesis, under which the benefits relate to increased care and paternal investment (Barash, 1977; Beach, 1974; Eibl-Eibesfeldt, 1970; Hamburg, 1978; Morris, 1999).

Genetic benefits to offspring can, in theory, be any heritable traits with fitness benefits, but the focus has been on physical masculinity and attractiveness, which are commonly assumed to reflect genetic quality in men (Gangestad & Scheyd, 2005; though see Lee et al., 2014). Several studies have found that orgasm rate is higher with more attractive partners (Andersson, 1994; Gallup, Ampel, Wedberg, & Pogosjan, 2014; Grammer, Fink, Møller, & Thornhill, 2003; Shackelford et al., 2000). Most relied on women's reports regarding her own partner, or women's reports of how attractive her friends would find her partner (Gallup et al., 2014; Shackelford et al., 2000), though Thornhill et al. (1995) and (Puts, Welling, et al., 2012) found the same effect with independent ratings of partners. Puts, Welling, et al. (2012) also found that highly orgasmic women's partners were rated as more dominant and masculine by online volunteers than were the partners of minimally orgasmic women.

Benefits conferred to offspring through paternal investment can include resource provision, physical protection, and infant care (Carter, 1992; Lloyd, 2005). Such benefits are more available when the father stays pair-bonded to the mother, so traits such as faithfulness and emotional warmth are often cited as important indicators of paternal investment, in addition to resource measures such as earning potential (Buss & Barnes, 1986; Gallup et al., 2014; Scheib, 2001). While the role of these traits in predicting orgasm has not been investigated thoroughly (though see Herberich, Hothorn, Nettle, & Pollet, 2010; Pollet & Nettle, 2009), Costa and Brody (2007) found that overall relationship quality was associated with greater orgasm frequency during penetrative sex. However, neither Zietsch et al. (2011) nor Thornhill et al. (1995) found a relationship between relationship commitment or length and orgasm frequency. In regard to resource provision, Gallup et al. (2014) observed that partners’ family income was predictive of orgasm frequency.

Many traits, however, such as intelligence, could provide offspring benefits through paternal investment (e.g. via higher earning potential) as well as genetic inheritance, so we do not consider all partner traits as dividing neatly into ‘good genes’ or ‘good dad’ categories. Nevertheless, to be usefully distinct hypotheses, they must yield differential predictions regarding the kinds of traits that will be possessed by partners with whom female orgasm rate is higher versus lower. Only one study has tested the association of women's orgasm frequency with a range of partner traits (Gallup et al., 2014). They found that women's orgasm frequency was predicted by partners’ attractiveness and family income. However, the study used a between-subjects design, which has two important limitations. First, power to detect associations between orgasm frequency and orgasm rates is reduced because the ‘noise’ of between-woman variation in orgasm frequency obscures the between-partner variation. Second, any associations that are found may be subject to confound – that is, highly orgasmic women may choose or attract or retain different partners than less-orgasmic women. For example, the dating site OkCupid found among 42,398 site users that women who enjoy exercise have markedly greater ease of orgasm (Rudder, 2011) – such women, being fitter and healthier, may also tend to partner with more attractive men.

To address these limitations, the present study used a within-subjects design. First, we asked to what extent women actually experience variation in orgasm rate between different partners. We then compared, in women who had multiple ex-partners, the traits of the partner with whom they experienced orgasms at the highest rate (‘high-orgasm partner’) against the partner with whom they experienced orgasms at the lowest rate (‘low-orgasm partner’).

Further, we measured the type of sexual activity engaged in with high-orgasm and low-orgasm partners. Women are more likely to achieve orgasm when their partners engage in other sexual activity prior to intercourse (Richters, de Visser, Rissel, & Smith, 2006) and when their clitoris is manually stimulated during sex (Hite, 1977). It could be that these activities distinguish high-orgasm and low-orgasm partners, rather than (or as well as) personal characteristics. This is the first study to test within-woman associations between orgasm frequency and variation in partner traits and sexual behaviour – associations that are key to assessing mate-choice hypotheses of female orgasm.

## Method

### Participants

In order to avoid bias in the evaluation of sexual partners, we recruited single women, as those in a relationship may feel obliged to rate their current partner more favourably than is strictly true. We therefore launched a screener survey to identify suitable participants to take part in the study. In order to qualify, participants were required to (1) be female, (2) not currently be in a relationship, (3) identify as heterosexual, and (4) have had more than two sexual partners in their lifetime. Initial screener surveys were launched on Amazon's Mechanical Turk. Participants were offered US$0.05 to complete the screener survey, with the possibility of a larger payment if they qualified for the main survey. This screener survey was completed by 1,069 participants, of whom 123 qualified. Of these, 103 participants completed the full survey. Participants earned $US3.50 for completing the full study. Participant age ranged from 20 to 69 (*M*=36.49, *SD*=12.19). Ninety-seven participants (93%) were from the United States, while six were from Australia, New Zealand, Great Britain, and Canada. The majority were Caucasian (81.6%). On average, participants took 71 min to complete the survey (note that the items used in the present study constitute one component of a larger survey exploring female sexuality and relationships).

### Procedure

Participants were informed that participation was voluntary and anonymous and that they could withdraw without penalty. Participants then answered questions relating to their ‘high-orgasm’ and ‘low-orgasm’ partners (counterbalanced). At the end of the survey, participants were asked if they had misrepresented any information. They were assured they would receive full payment even if they had. Fourteen women indicated that they were in a relationship at the time of the survey. These women's data were included when analysing general sexual behaviour, but excluded when comparing high- and low-orgasm-inducing partners to prevent current partner bias. Participants were debriefed at the end of the study.

### Measures

Participants were assessed on their general sexual preferences and behaviours as well as their sexual behaviour with a partner with whom they orgasmed easily and one with whom orgasm was difficult or absent. Additionally, participants answered a series of items regarding the characteristics of the partner and their relationship.

#### Demographics

Participants recorded basic demographic details such as their age, height, weight, and ethnicity. Relationship status and duration (where applicable) were assessed to detect partnered participants.

#### General sexual behaviour

To investigate variation in orgasm frequency, participants were first asked to report the frequency of self and partner clitoral stimulation duration during intercourse. Participants were then asked to indicate their general orgasm frequency (i.e. not with a particular partner) during sex without manual clitoral stimulation, sex with partner clitoral stimulation, and sex with self-stimulation of the clitoris. Finally, for each measure of orgasm frequency during intercourse, participants were asked to what extent their orgasm frequency changed depending on the partner. Frequency of orgasm on all items ranged from 1 (‘never’) to 6 (‘always’) while variation in orgasm ranged from 1 (‘always the same, doesn't depend on who I'm with’) to 4 (‘very different depending on who I'm with’).

#### Partner characteristics

All participants then completed two identical partner characteristics sections concerning a high-orgasm partner and a low-orgasm partner (counterbalanced between participants). Participants were asked to think of a partner with whom they had orgasmed the most easily during sex (high-orgasm) and a partner with whom they had the most difficulty orgasming during sex (low-orgasm). If participants reported no variation in their orgasm frequency, they were asked to describe their most recent and second most recent sexual partner instead of high and low. These data were later excluded from partner comparison analysis (see Results). Participants were then asked for the duration of the relationship and how long ago they last slept with this partner. This was done in order to control for the possibility that women might be biased to regard more recent (or earlier) partners more positively. Participants were then asked to rate this partner on the following traits ranging from 1 (‘much lower than average’) to 5 (‘much higher than average’).

Traits were selected based on previous claims of association with parental quality (i.e. faithfulness, warmth, earning potential, and kindness; Buss & Barnes, 1986; Pollet & Nettle, 2009; Scheib, 2001) or genetic quality (i.e. physical attractiveness (Prokop & Fedor, 2011; Puts, Welling, et al., 2012), height (Prokop & Fedor, 2011), athleticism (Schulte-Hostedde, Eys, & Johnson, 2008), muscularity (Frederick & Haselton, 2007; Lassek & Gaulin, 2009), voice depth (Puts, 2005; Puts, Gaulin, & Verdolini, 2006), physical fitness (Schulte-Hostedde et al., 2008), humour (Simpson & Gangestad, 1992), creativity (Haselton & Miller, 2006; Miller, 2000), intelligence (Haselton & Miller, 2006; Miller, 2000), dominance (Simpson & Gangestad, 1992), and body odour pleasantness (Garver-Apgar, Gangestad, Thornhill, Miller, & Olp, 2006; Wedekind, Seebeck, Bettens, & Paepke, 1995)). Women also reported partners’ facial hair (as a masculine trait that is easily changeable and therefore not likely to be an indicator of genetic quality), as well as their partners’ confidence, weight, penis length, and penis width. Participants were also asked to judge how attractive their friends found this partner on the same scale as other partner traits, based on findings from Sela, Weekes-Shackelford, Shackelford, and Pham (2015) and Gallup et al. (2014).

#### Partner sexual behaviour

Participants then described the sexual behaviour of their high- and low-orgasm partners immediately after rating their characteristics. Participants were asked how frequently they discussed sexual positions with their partner (1 ‘never’ to 5 ‘very often’) and whether they had asked their partner to use specific positions to increase the likelihood of reaching orgasm (yes/no). The frequency of sexual activities that did not involve penetrative sex, specifically receiving oral sex, use of sex toys, and dirty talk was then assessed (1 ‘never’ to 5 ‘always’) given their possible relationship with orgasm rates. Participants were then asked to indicate the time taken in minutes (1) spent on foreplay with this partner, (2) for this partner to reach orgasm, and (3) for the participant to reach orgasm with this partner. As orgasm and clitoral stimulation can vary based on sexual position, a series of seven cartoon depictions of common sexual positions were presented and participants were asked to indicate the frequency of orgasm and self and partner manual clitoral stimulation (1 ‘never’ to 5 ‘always’) in each. The positions were face-to-face man above, face-to-face woman above, face-to-face side position, face-to-face sitting position, rear-entry prone position, rear-entry kneeling position, and rear-entry sitting position.

#### Relationship variables

Last, participants were asked to indicate if the following statements were true or false for each of the partners they described. These included: ‘this is actually a current partner’, ‘the sex I described was non-consensual’, ‘I had sex with this partner fewer than five times’, and ‘this was an adulterous encounter’.

## Results

### General sexual practices

Women reported variation in orgasm during intercourse based on different sexual behaviours (see Table 1 for descriptive statistics). Orgasm frequency was significantly lower during intercourse without manual clitoral stimulation (*M*=2.75, *SD*=1.31) when compared to intercourse paired with self-stimulation of the clitoris (*M*=3.97, *SD*=1.39), *t*(90)=−7.70, *p*<0.001, and partner stimulation of the clitoris (*M*=3.89, *SD=*1.28), *t*(96)=−7.09, *p*<0.001. However, no significant differences were observed in orgasm frequency when comparing intercourse paired with self- and partner stimulation, *t*(85)=1.90, *p*=0.061.

**Table 1:** General sexual behaviour descriptive statistics

Question	*N*	Range	M	SD
Age	102	20–69	36.49	12.19
Frequency of self-stimulation during intercourse (1=Never, 6=Always)	103	1–6	2.97	1.14
Frequency of orgasm using self-stimulation during intercourse (1=Never, 6=Always)	91	1–6	3.97	1.39
Rate of change in orgasm using self-stimulation during intercourse (1=Always the same …, 4=Very different….)	91	1–4	2.64	0.91
Frequency of partner stimulation during intercourse (1=Never, 6=Always)	102	1–6	3.38	1.09
Frequency of orgasm using partner stimulation during intercourse (1=Never, 6=Always)	97	1–6	3.89	1.28
Rate of change in orgasm using partner stimulation during intercourse (1=Always the same…, 4=Very different….)	96	1–4	2.80	0.89
Frequency of orgasm during intercourse without manual stimulation (1=Never, 6=Always)	103	1–6	2.79	1.31
Rate of change in orgasm during intercourse without stimulation (1=Always the same…, 4=Very different…)	103	1–4	2.41	1.05
Orgasm frequency during vaginal only masturbation (1=Never, 6=Always)	48	1–6	3.63	1.41
Clitoral and vaginal stimulation during masturbation (1=Not at all, 4=Always)	99	1–4	2.18	0.84
Orgasm frequency during both vaginal and clitoral masturbation (1=Never, 6=Always)	80	1–6	5.09	1.17

Women reported wide variation in their orgasm frequency during intercourse. Variation in orgasm also differed based on the type of sexual intercourse. Women reported that their orgasms varied more between partners when self-stimulating of the clitoris during intercourse (*M*=2.64, *SD*=0.91) and with partner stimulation of the clitoris (*M=*2.80, *SD*=0.89), when compared to sex without manual clitoral stimulation (*M*=2.38, *SD*=1.05), *t*(90)=2.06, *p*=0.042 and *t*(95)=2.96, *p*=0.004. However, females reported greater variability in their orgasms between partners when their partner was stimulating their clitoris compared to their own stimulation, *t*(84)=2.86, *p*=0.005.

### Sexual partner characteristics

Six women indicated that one of their partners was non-consensual, and 18 (17%) could not distinguish between a partner with whom they orgasmed easily or one with whom they had difficulty achieving orgasm. After excluding these data points, the final sample contained information regarding the high- and low-orgasm partners of 71 women. If a participant-reported time to orgasm with a partner was greater than 120 min (1: 1.4%), it was assumed that she did not orgasm with this partner and this response was removed. As a manipulation check, we then compared reported orgasm frequency between high- and low-orgasm males across all sexual positions. As expected, participants were more likely to orgasm in every position with a high-orgasm male (*p*≤0.002). We subsequently averaged orgasm frequency over all sexual positions and compared this between high and low partners. Again, orgasms were more frequent for high-orgasm partners on average, *t*(69)=7.591, *p*<0.001. Further comparisons of high and low partners were separated into partner characteristics (Table 2) and sexual behaviour with these partners (Table 3).

**Table 2:** Comparing most orgasmic and least orgasmic partners on personal characteristics

Trait	High-orgasm partner M(SD)	Low-orgasm partner M(SD)	*t*	*df*	*p*	*d*
Humour	3.72 (0.91)	3.28 (0.96)	3.497	70	0.001*	0.42
Intelligence	3.55 (0.97)	3.27 (0.93)	1.944	70	0.056	0.23
Dominance	3.10 (0.90)	2.96 (1.03)	0.890	69	0.377	0.10
Attractiveness	3.59 (0.87)	3.04 (0.84)	4.186	70	<0.001*	0.50
Friends find partner attractive	3.27 (0.93)	3.00 (0.85)	2.036	69	0.046	0.24
Athleticism	2.91 (0.99)	2.71 (0.97)	1.342	69	0.184	0.16
Creativity	3.62 (1.03)	2.63 (0.87)	7.630	70	<0.001*	0.92
Muscularity	3.11 (0.96)	2.79 (0.81)	2.266	70	0.027	0.27
Voice depth	3.11 (0.69)	2.96 (0.66)	1.496	70	0.139	0.17
Fitness	3.08 (0.94)	2.82 (0.88)	1.907	70	0.061	0.22
Emotional warmth	3.40 (1.04)	2.77 (1.02)	3.797	69	<0.001*	0.46
Faithfulness	3.37 (1.23)	2.65 (1.17)	3.771	70	<0.001*	0.45
Earning potential	3.23 (1.06)	2.85 (1.12)	2.400	70	0.019	0.26
Height	3.51 (0.86)	3.29 (0.76)	1.635	69	0.107	0.19
Weight	3.06 (0.77)	3.03 (0.70)	0.270	70	0.788	0.03
Penis length	3.34 (0.81)	2.97 (0.94)	2.714	70	0.008	0.33
Penis width	3.39 (0.80)	2.97 (0.89)	3.019	70	0.004	0.36
Confidence	3.66 (0.86)	3.20 (1.02)	3.158	70	0.002	0.37
Kindness	3.48 (1.03)	2.96 (0.87)	3.303	70	0.002	0.39
Body odour pleasantness	3.66 (0.93)	3.20 (0.94)	3.626	70	0.001*	0.42
Facial hair	1.89 (1.13)	1.87 (1.01)	0.083	70	0.934	0.01

* Indicates significance at Bonferroni corrected *p*≤0.001.

**Table 3:** Comparing most orgasmic and least orgasmic partners on sexual behaviour

Behaviour	High-orgasmic partner M(SD)	Low-orgasmic partner M(SD)	*t*	*df*	*p*	*d*
Length of relationship (months)	31.35 (38.85)	22.07 (30.40)	1.688	70	0.096	0.20
Time since last intercourse (months)	80.93 (107.37)	82.13 (94.97)	−0.121	70	0.904	−0.01
Age during last intercourse (years)	30.78 (9.59)	30.64 (10.76)	0.162	69	0.872	0.02
Frequency of communicating position preferences (1–5)	3.04 (1.01)	2.20 (0.99)	6.095	68	<0.001*	0.73
Pleasure focused (1–5)	3.93 (0.92)	2.54 (1.07)	8.339	70	<0.001*	0.94
Frequency of receiving oral sex (1–6)	3.70 (1.53)	2.69 (1.31)	4.425	70	<0.001*	0.53
Use of sex toys (1–6)	1.77 (1.22)	1.27 (0.70)	3.579	70	0.001*	0.45
Use of dirty talk (1–6)	2.77 (1.35)	2.25 (1.24)	3.108	70	0.003	0.37
Minutes of foreplay	17.63 (11.36)	10.01 (9.97)	4.869	70	<0.001*	0.58
Minutes to orgasm (partner)	17.11 (11.87)	12.07 (14.44)	2.547	70	0.013	0.30
Minutes to orgasm (female)	16.63 (10.72)	26.55 (61.28)	−1.526	65	0.132	−0.29
Partner clitoral stimulation averaged over all positions (1–6)	2.44 (1.05)	1.71 (0.69)	6.174	69	<0.001*	0.78
Self-clitoral stimulation averaged over all positions (1–6)	2.21 (0.94)	1.80 (0.92)	3.733	69	<0.001*	0.42

* Indicates significance at Bonferroni corrected *p*≤0.001.

#### Partner traits

Partner humour, voice depth and facial hair length were substantially skewed and as such we first conducted non-parametric tests comparing high and low partners on these traits. Wilcoxon signed-rank tests indicated that high-orgasm partners were rated significantly higher on humour than low-orgasm males (Z=3.16, *p*=0.002), but no differences were observed in vocal depth (Z=1.43, *p=*0.154), or facial hair (Z=0.01, *p*=0.990). As these results did not differ from parametric tests, we report parametric results in Table 2 for ease of interpretation. Following Bonferroni corrections for multiple comparisons (34 in total), paired t-tests indicated that a number of traits and behaviours differed between high- and low-orgasm partners at *p*≤0.001. With this criteria, humour, attractiveness, creativity, emotional warmth, faithfulness, and body odour pleasantness were all significantly greater in high-orgasm partners (see Table 2).

#### Partner sexual behaviours

Length of relationship, length of time since last sexual encounter, and age at last sexual encounter were all substantially skewed. As a result, non-parametric tests were used for preliminary analysis. No significant differences emerged between length of relationship with high- and low-orgasm partners (Z=1.46, *p=*0.145), length of time since the last sexual encounter (Z=0.56, *p*=0.576), nor age during the last sexual encounter (Z=0.60, *p*=0.548). These results were no different from those observed using paired t-tests, which we report for ease of interpretation (Table 3). A McNemar's paired samples test of dichotomous outcomes indicated that females were no more likely to have had sex with high- or low-orgasm partners fewer than five times, *p*=0.388, nor were they more likely to have had an affair with either partner, *p*=0.999.

A number of sexual behaviours differed significantly between high- and low-orgasmic partners (see Table 3). Females in the sample communicated with high-orgasm partners about sexual positions more frequently and also received oral sex from high-orgasm partners more frequently. High-orgasm males were also reported as having a greater focus on female pleasure. In addition, high-orgasm partners were more likely to use sex toys and spend more time on foreplay. Averaged over all sexual positions, high-orgasm males were also more likely to manually stimulate the clitoris. Similarly, females were more likely to stimulate their own clitoris with high-orgasm males.

## Discussion

We collected data from sexually active females regarding their general sexual activity. We also collected their reports regarding a partner with whom orgasm came the most easily and one with whom orgasms were the most difficult to achieve. Our aim was to determine whether women were conscious of orgasm variation with different partners, whether this variation was related to mate characteristics, and whether this variation was related to different sexual practices. First, women reported (on average) variation in personal orgasm frequency between partners. Variation in orgasm frequency was also significantly greater when manual clitoral stimulation occurred. Females reported that their orgasm frequency varied more between partners when partners stimulated their clitoris, compared to when they stimulated themselves. This suggests that orgasm variation is contingent upon sexual skill in addition to mate characteristics. This group of findings represent the first attempt to determine if women report orgasming more or less with different partners. Our results show that they do, and thus allowed us to be confident when moving on to between-partner traits and behaviours that may account for this difference.

After comparing the characteristics of high- and low-orgasm partners, a number of differences emerged in both individual characteristics and sexual behaviour. On average, high-orgasm male partners were more humorous, attractive, creative, emotionally warm, faithful and had more pleasant body odour than low-orgasm partners. These findings are somewhat consistent with previous research and with aspects of the mate-choice hypothesis. High-orgasm males were higher in characteristics associated with parental investment. Emotional warmth and faithfulness are both factors that may contribute to pair bonding and may encourage a greater number of copulations within pairings as well as investment in any subsequent offspring (Buss & Barnes, 1986; Pollet & Nettle, 2009; Scheib, 2001). These traits have not previously been identified in high-orgasm partners, including Costa and Brody (2007) who observed that commitment, a similar relationship component, was unrelated to sexual satisfaction in a small sample of women in relationships.

High-orgasm partners were also, on average, higher in a number of characteristics that may be construed as indicative of both genetic benefits and parental investment. Both attractiveness (Mitchem et al., 2014) and creativity (e.g. musicality: Mosing et al., 2014; Trainor, Honing, Peretz, Gingras, & Fisher, 2015) are heritable traits and may confer genetic benefits to offspring (e.g. Miller, 2000).

In agreement with Gallup et al. (2014), we also observed that males with whom orgasms were more frequent were rated as more humorous than low-orgasm partners. In Gallup et al. (2014), ratings of partners humour were also associated with higher ratings of creativity and intelligence and indeed high-orgasm males in the present study were also considered to be more creative than low-orgasm males. It has been proposed that humour and creativity may act as an honest signal of intelligence (Miller, 2000) and therefore an indicator of genetic quality. Intelligence can be more difficult to gauge than humour and creativity, with only small to moderate correlations between perceived and actual intelligence scores (Borkenau, Mauer, Riemann, Spinath, & Angleitner, 2004). Meanwhile, humour and creativity are more naturalistically demonstrated in dynamic, social contexts and as such may offer a more reliable indication of intelligence.

Body odour pleasantness has also been implicated in mate choice previously and may be associated with genetic benefits. After rating the pleasantness of body odour from shirts worn by males in the previous 48 h, it was found that females preferred the shirts of males with dissimilar immune system alleles (Wedekind et al., 1995). Not only are key components of immune function heritable (de Craen et al., 2005), but women also report lower sexual satisfaction with partners who share the same immune system alleles (Garver-Apgar et al., 2006). It has been hypothesised that selecting mates with dissimilar immune system alleles may confer benefits to offspring as they may inherit a wider range of antibodies (Havlicek & Roberts, 2009).

While the above findings may represent partial support for aspects of mate-choice hypotheses, some aspects of the findings conflict with the sire-choice hypothesis. While we found attractiveness (note that this may or may not have been interpreted by participants to refer to *physical* attractiveness) was rated higher in high-orgasm partners, we found no significant difference, after correcting for multiple comparisons, between high- and low-orgasm partners in terms of putative ‘good genes’ traits such as intelligence, athleticism, and fitness, nor sexually dimorphic traits such as height, dominance, muscularity, and voice depth. The pattern found in our results suggests, conversely, that women's orgasms depended more on traits potentially representing investment and attentiveness (e.g. faithfulness, emotional warmth) than classic markers of good genes and masculinity. Potentially more important, as will be discussed below, were what these attractive, creative, warm, and faithful partners were reported to have done in bed.

Sexual behaviour varied considerably between high- and low-orgasm partners. During intercourse, high-orgasm males were more likely to focus on female pleasure, communicate about sexual positions, use sex toys, and perform oral sex on the females in this sample. On average across all sexual positions, high-orgasm partners were also more likely to stimulate their partner's clitoris and women appeared to be more comfortable stimulating their own clitoris with high-orgasm partners (potentially as a result of communication and high-orgasm partner's focus on their sexual pleasure). The only two domains in which behaviour did not differ between high- and low-orgasm partners were the use of dirty talk, and length of time the male partner took to reach orgasm.

The study was not without limitations. For example, it is difficult to discern the relative independent contribution of partner characteristics to the female orgasm. For instance, greater levels of humour and creativity may simply contribute to ratings of attractiveness, which in turn increases the likelihood of orgasm. Moreover, high-orgasm partners engaged in behaviours that were more likely to elicit an orgasm than their low-orgasm counterparts, and it is unclear how this relates to the personal characteristics of those partners. We find that women report greater orgasm variation between partners when their clitoris is manually stimulated compared to when it is not. This finding hints at the fact that some men might be particularly adept at inducing orgasm during penetrative sex via clitoral stimulation relative to others. Further, it seems that women themselves are effective at inducing orgasm via self-stimulation of the clitoris more so with some partners than others. It should be noted that clitoral stimulation appears to be very important when considering between-partner ease of orgasm in general, given that high-orgasm males were more likely to stimulate the clitoris than low-orgasm males. A future investigation of variation in orgasm frequency between high- and low-orgasm males may consider examining sex with and without clitoral stimulation separately to establish whether there are unique factors that predict female orgasmability in the absence of direct exterior clitoral stimulation.

Studies of female orgasm requiring self-report of sexual behaviour and partner characteristics can also be biased by ‘halo’ effects – either orgasm frequency may cause women to overestimate their partner's other positive qualities or conversely, their partner's positive qualities may cause them to overestimate their orgasm frequency. However, we do not believe this presents a substantial issue in the present study, as many socially desirable traits were not found to significantly differ between high- and low-orgasm males. For instance, intelligence is a highly socially desirable trait that is associated with numerous beneficial outcomes. Likewise, height and earning potential are substantially important in female attraction yet neither differed significantly between high- and low-orgasm partners.

Future research should also aim to replicate our findings, and compare the unique predictive strength of each characteristic. Furthermore, meaningful mediational paths might emerge. For example, emotionally warm men might focus more on women's pleasure, stimulating their clitoris more, and thus eliciting more orgasms.

Overall, however, our findings give some support to the idea that the female orgasm helps to choose partners who are likely to be good fathers, but suggest scepticism is warranted regarding the sire-choice hypothesis, which emphasises genetic benefits to offspring. It also raises the previously ignored complication that variation in specific sexual behaviours plays at least as much of a role as do the partners’ personal characteristics in how effectively he induces orgasms.

At a broader level, mate-choice hypotheses of female orgasm raise two questions. First, how and why are the clitoris and vagina (the stimulation of which produces orgasm) necessary or even useful for assessing partner quality? Why not just rely on other sensory apparatus and a massive brain? Second, an orgasm is a short on/off pleasure burst – what does this add to mate assessment over and above a smooth gradient of pleasure (e.g. the higher the partner quality, the greater the pleasure)? Clarification of these basic theoretical questions, as well as further empirical work in the direction of the present study, should help assess the viability of mate-choice hypotheses of female orgasm.
